# Media consumption and youth work competencies: Evidence from the Hong Kong youth survey

**DOI:** 10.1371/journal.pone.0349087

**Published:** 2026-05-14

**Authors:** Boris Lok-Fai Pun, Jeremy Ko, Anthony Ying-Him Fung, Janice Ka-Yee Wong

**Affiliations:** 1 Hong Kong Institute of Asia-Pacific Studies, Chinese University of Hong Kong, Hong Kong; 2 Center for Comparative and International Studies, ETH Zurich, Switzerland; 3 Faculty of Social Science, Chinese University of Hong Kong, Hong Kong; 4 School of Communication, The Hang Seng University of Hong Kong, Hong Kong; Universiti Malaya, MALAYSIA

## Abstract

This study examines how the number of hours spent on different types of media consumption is associated with youth work‑readiness competencies in Hong Kong. Using survey data from 363 tertiary students across six institutions (October to December 2024), the analysis investigates relationships between weekly hours of exposure to six media forms—newspapers, online media, television, social media, content‑creator channels, and online forums—and three dimensions of work readiness: workplace soft skills, reflection and learning, and career aspiration/planning. Results show that structured and feedback‑rich media, such as newspapers and purposeful social media use, are positively associated with work‑readiness competencies, while unstructured or entertainment‑driven media, including online forums and content‑creator channels, are negatively associated. These findings suggest that not only the quantity but also the quality and structure of media engagement influence the development of key competencies. The study highlights that structured media environments serve as important contexts for learning and skill formation among Hong Kong youth.

## 1. Introduction

Hong Kong’s transition into an advanced service‑ and knowledge‑based economy has intensified concerns about youth preparedness for the labor market. Despite the city’s reputation as a global financial and innovation hub, younger generations continue to face persistent barriers to stable employment and career progression. The unemployment rate for persons aged 20–24 stood at 8.38 percent in 2024—more than double the overall rate of 3.1 percent [1]. At the same time, median wages for those aged 15–34 have grown more slowly than those of older workers, narrowing prospects for advancement [2]. Hong Kong’s post‑handover development reflects a dual structure: while the economy increasingly depends on creativity and knowledge‑intensive industries, income distribution remains polarized and occupational advancement has stagnated [3,4]. Within this polarized context, youth work readiness—particularly the development of transferable and adaptive competencies—has become a central concern for employability and long‑term economic resilience.

As one of the world’s most digitally connected societies, Hong Kong provides a unique setting for examining how media environments shape youth competencies. With digital and social platforms integrated into almost every aspect of daily life, media now function not only as channels for communication but also as learning environments where young people encounter, interpret, and negotiate ideas about work, professionalism, and success [5–7]. Official statistics show that more than 96 percent of households have internet access and over 95 percent of individuals aged 15–24 use smartphones daily, spending on average more than three hours per day on social media [8]. This level of connectivity suggests that media constitute an important environment in which young people in Hong Kong develop habits of learning, reflection, and communication that may be associated with their work‑readiness competencies [9].

However, high digital connectivity does not necessarily correspond with skill‑building or professional development. Research has shown that online participation among Hong Kong youth tends to be cautious and consumption‑oriented rather than participatory or creative [5]. Many young people are digitally proficient but prefer private communication and selective engagement, often “watching but not voicing.” Instead of producing or exchanging original content, they use digital media mainly for social interaction, leisure, or information following, avoiding public commentary due to reputational concerns. This pattern reflects an ambivalent digital citizenship—high connectivity and media fluency coexisting with limited active engagement and skill application [5]. These consumption‑oriented behaviors are significant because they highlight how media function as social learning contexts that can either reinforce or undermine the formation of work‑related competencies. Structured and feedback‑rich media—such as newspapers or professional social media platforms—may cultivate critical thinking, reflection, and communication skills, while unstructured or entertainment‑driven media—such as influencer channels or online forums—may provide little reinforcement for cognitive or interpersonal development [10–12]. Understanding how different types of media, and the number of hours youth devote to them, are associated with specific aspects of work‑readiness competencies is therefore essential for explaining how young people prepare for employment in a digitally saturated environment.

Recognizing these dynamics, this study examines the associations between the number of hours spent consuming different types of media and youth work‑readiness competencies in Hong Kong. It focuses on tertiary students—a cohort situated at the threshold between higher education and labor‑market entry—whose media habits and learning behaviors offer insight into how media use relates to the development of employability‑related skills. The analysis considers six forms of media—newspapers, online media, television, social media, content‑creator channels, and online forums—and their associations with three key dimensions of work readiness: (1) workplace soft skills, (2) reflection and learning, and (3) career aspiration/planning. These competencies represent the behavioral, cognitive, and motivational foundations that equip young people to navigate transitions from education to employment in Hong Kong’s service‑ and knowledge‑based economy [2,13,14].

Accordingly, the paper proceeds as follows: Section 2 reviews theoretical and empirical research linking media consumption and youth work competencies; Section 3 outlines the research design and methods; Section 4 presents key findings; and Section 5 discusses implications, limitations, and directions for future research.

## 2. Literature review

### 2.1. Interplay between structural conditions, personal agency, and career competencies in youth mobility

Work readiness and employability reflect the interaction between structural conditions and individual agency. Research shows that socioeconomic disparities, educational stratification, and limited occupational diversification continue to influence young people’s career outcomes [15]. In modern economies, these structural barriers often coincide with intensified competition, which reinforces the importance of developing strong personal competencies for navigating career transitions [16]. At the same time, empirical findings underscore that personal cognition—such as self‑efficacy, outcome expectations, and goal orientation—plays a crucial role in how individuals respond to external challenges [17,18]. This perspective aligns with Social Cognitive Career Theory (SCCT), which emphasizes the dynamic interplay between personal agency and environmental influences [19–21]. According to SCCT, contextual systems such as schools, workplaces, and media environments expose individuals to behavioral models, social feedback, and evaluative mechanisms that shape perceptions of competence and attainable goals. Through this continual interaction, people develop self‑efficacy (belief in one’s abilities to perform tasks effectively) and outcome expectations (beliefs about the likely results of one’s actions). These beliefs, in turn, influence how individuals pursue learning opportunities and adapt to professional demands.

Within this framework, work competencies can be understood as the integration of behavioral, cognitive, and interpersonal capacities necessary for effective performance in professional contexts. They encompass a range of attributes—such as communication, collaboration, reflection, continuous learning, and career goal‑setting—that translate potential into actual employability [22]. Repeated mastery experiences—such as problem solving, teamwork, and structured reflection—enhance self‑efficacy by reinforcing a sense of control and capacity to succeed [9,23]. Meanwhile, positive environmental cues—such as feedback, mentorship, and exposure to professional exemplars—strengthen efficacy beliefs and adaptive expectations [9,24]. Studies in Hong Kong and comparable contexts [9,25] indicate that youth who possess higher self‑efficacy and stronger work competencies demonstrate higher confidence in managing career uncertainties and transitions. Under this view, the environment serves both as a learning and interpretive context: it provides behavioral models, reinforces performance through observation, and offers evaluative feedback that facilitates competence building. When young people engage purposefully with such environments, they convert structural exposure into internalized skills and actionable capacities for career progression [26].

### 2.2. Media usage and career related skills development

As youth increasingly engage with diverse media environments, these platforms have become central to career learning and competency development. In line with SCCT, media can be regarded as environmental systems that provide social, cognitive, and motivational cues influencing how individuals acquire and apply work‑related skills. Media do not simply transmit information but also model professional behaviors, problem‑solving strategies, and communicative norms that underpin employability [27]. Studies emphasize that active and purposeful engagement with structured media fosters competencies essential for workplace readiness—such as critical thinking, digital collaboration, and self‑reflection [28,29]. Traditional media, particularly newspapers and structured news platforms, reinforce analytical reasoning and cognitive discipline that strengthen professional communication and continuous learning [11]. Digital spaces, especially social media, offer collaborative and interactive environments that simulate professional settings and enable verbal persuasion through feedback and discussion [34]. However, unstructured or entertainment‑oriented media—such as informal online forums or influencer‑driven content channels—often lack consistent reinforcement or learning feedback, which may hinder focused cognitive engagement [10,12]. Collectively, these findings suggest that media consumption is associated with youth competency development through mechanisms of observation, reflection, and reinforcement, consistent with SCCT’s explanation of environmental learning pathways.

While the evidence base linking technology and employment outcomes is expanding, most studies have examined digital access or infrastructure rather than psychological and cognitive development. For instance, Weldeyesus and Alemu (2024) [30] investigated mobile phone access and labor participation in Sub‑Saharan Africa, Studies in developed contexts are typically oriented toward visibility and branding: McGrath (2023) [31]  explored social media self‑presentation among young professionals in the United States, while Masterson (2021) [32]  examined influencer culture and entrepreneurial aspirations in the United Kingdom. These studies enrich understanding of media‑based career behaviors but do not comprehensively address how different types of media consumption relate to distinct dimensions of employability competencies.

Furthermore, while previous research has separately examined youth employment challenges [15,16] and skill development [9,23], relatively few integrate these aspects within a unified theoretical framework grounded in environmental learning and individual agency. For example, Fung et al. [9] analyzed work competencies and career readiness among Hong Kong youth but did not assess media environments as learning contexts, while Zhang and Huang (2025) [25] investigated self‑efficacy development in Chinese settings without addressing media exposure. Likewise, Wu and Jung [17] examined agency development among Asian youth but focused on familial or educational contexts rather than digital systems. This fragmentation underscores a need for studies that examine how informational environments, particularly media, contribute to the behavioral and cognitive foundations of career preparedness. This gap is especially salient in high‑connectivity societies like Hong Kong, where young people navigate complex informational ecosystems while facing competitive labor markets. The present study addresses this gap by examining the associations between media engagement and the development of work‑readiness competencies within an SCCT‑based framework. Specifically, it investigates how time spent across six media categories—newspapers, online media, television, social media, online forums, and content‑creator channels—relates to three key domains of work readiness: (1) workplace soft skills, (2) reflection and learning, and (3) career aspiration and planning. This two‑stage analytical approach clarifies whether different patterns of media consumption align with specific competency profiles, thereby extending understanding of how informational and social learning environments contribute to youth readiness for employment in technologically saturated societies.

### 2.3. Proposed mechanism and research hypothesis

In this study, the distinction between structured and unstructured media is analytically predefined rather than inferred from the results. While SCCT does not classify environments by structure, it emphasizes that efficacy development depends on the quality of learning opportunities—specifically, the availability of consistent feedback, clear goals, and opportunities for reflection and reinforcement [21]. Building on this logic and prior media‑learning research [e.g., 10,11], structured media are defined as environments with editorial or moderation control, higher cognitive demand, and clear feedback mechanisms that support sustained, purposeful engagement (e.g., newspapers or professionally oriented social platforms). Unstructured media, such as entertainment‑driven channels or unmoderated online forums, lack these features, offering fragmented or episodic content and minimal reinforcement, and thus provide weaker conditions for reflection or skill integration. This a priori conceptualization guided both the operational coding of media types and the interpretation of the results. Within this theoretical framework, media are understood as environmental contexts through which individuals observe competence, interpret professional norms, and build confidence in their own capabilities. The degree of structure and feedback inherent in each medium shapes how effectively these experiences contribute to self‑efficacy and skill acquisition. As competencies develop through purposeful engagement and cognitive reinforcement, individuals become better prepared to manage workplace communication, problem‑solving, and decision‑making. Accordingly, the following hypotheses specify the expected relationships between different types of media consumption and youth work competencies (H1–H6). This analytical focus examines whether the patterns of associations are consistent with the theoretical framework that media exposure relates to competency development through environmental learning, observation, and reinforcement.

Engagement with traditional print media, particularly newspapers, develops self‑regulation, analytical thinking, and cognitive discipline—core components of self‑efficacy emphasized in SCCT [21]. Reading newspapers requires sustained attention and evaluative judgment, which encourages critical thinking and reflective argumentation—skills directly relevant to communication and problem‑solving in professional environments. These mastery experiences simulate structured work behaviors such as information selection, synthesis, and evidence‑based decision‑making [11]. Moreover, repeated exposure to professional discourse models normative behavior and occupational expectations, providing vicarious learning opportunities for professional conduct and reasoning. As readers internalize these cognitive patterns and linguistic styles, they strengthen their perceived capability to engage effectively in communication and problem‑solving in real‑world contexts. Over time, structured print engagement strengthens behavioral capability, fostering greater workplace readiness and self‑efficacy aligned with SCCT principles [19].

**H1:** Higher levels of newspaper consumption are positively associated with greater work competencies among youth.

In contrast, exposure to general online media delivers a large volume of information but often without adequate reinforcement or structured feedback, thereby weakening the self‑efficacy formation processes described by SCCT. While online media may provide cognitive stimulation, their fragmented and algorithmically driven content rarely offers the consistent patterns required for mastery learning or outcome validation [10,12]. Lent and Brown (2013) [33]  observe that environmental experiences that lack reinforcement interfere with the development of persistent goal pursuit. Under these conditions, information is often absorbed passively, unaccompanied by reflection or applied problem‑solving. Without structured modeling of competence, youth struggle to transform this fragmented stimulation into integrated skills relevant to the workplace. As a result, online media consumption may expand informational breadth but fail to strengthen the behavioral and cognitive self‑regulation necessary for sustained competency growth [27].

**H2:** Online media consumption shows no significant association with work competencies among youth.

Traditional television serves largely as an observational learning platform, yet it rarely supports the experiential reinforcement required for strong self‑efficacy development. SCCT posits that personal agency emerges most effectively when individuals engage in active mastery experiences and receive performance feedback [21,33]. Televised information typically positions viewers as passive recipients rather than active participants, lacking the iterative feedback systems—such as problem‑solving or dialogue—that are needed to translate observation into behavioral capability. Although television may convey social or occupational imagery, its limited opportunities for interaction prevent the conversion of symbolic observation into self‑belief and efficacy [10]. Over time, the absence of participatory reinforcement constrains television’s role as an environmental condition for cognitive skill acquisition. Therefore, consistent with SCCT’s assumption that self‑efficacy is built through active engagement and validation [19], the environmental contribution of television to skill formation remains minimal, aligning with prior findings that passive or entertainment‑driven media promote limited work‑related learning [12].

H3: Television consumption shows no significant association with work competencies among youth.

Social media platforms can potentially improve work readiness among youth because, when used purposefully, these platforms function as dynamic learning environments that promote social modeling, feedback exchange, and experiential learning. Within the SCCT framework, social media provide opportunities for vicarious learning and verbal persuasion—key mechanisms that strengthen self‑efficacy and support the development of essential professional attributes [19,34]. By engaging with professional networks, accessing career‑related content, and participating in collaborative discussions, young users can observe successful workplace behaviors, practice communication and problem‑solving skills, and receive feedback from peers and mentors. These experiences enhance cognitive engagement, adaptability, and confidence in applying learned skills to professional contexts [20].

Despite the unstructured and comparison‑driven nature of social media environments, such platforms can still contribute positively to competency formation when individuals engage with them intentionally and constructively. Although continuous exposure to rapidly changing content may fragment attention and limit sustained focus [27,34], purposeful participation—such as following professional networks, engaging in knowledge‑sharing communities, and reflecting on feedback—can transform these digital spaces into active sources of observational learning and self‑efficacy development. Even within unregulated or socially competitive settings, the breadth of perspectives, peer interactions, and real‑time feedback available can reinforce goal‑directed behavior, cognitive flexibility, and adaptive learning [7,10,29]. Consequently, while social media inherently carry risks of distraction, they possess substantial potential to function as informal yet powerful contexts for self‑regulated learning and skill advancement.

H4: Higher levels of social media consumption are positively associated with greater work competencies among youth.

Consumption of content‑creator or entertainment channels often emphasizes passive entertainment rather than structured skill‑building activities. Such content typically lacks the performance modeling, mastery experiences, and constructive feedback necessary for strengthening work‑related competencies according to SCCT principles [12]. Unlike educational or professionally oriented media, entertainment‑focused channels rarely provide opportunities for vicarious learning of workplace behaviors or the practice of cognitive and interpersonal skills. Without opportunities for observational learning of professional conduct or reinforcement of goal‑directed behavior, entertainment media consumption may divert time and attention from activities that build self‑efficacy and competence. As a result, individuals who spend substantial time-consuming entertainment content may develop fewer work‑related skills and demonstrate lower confidence in their professional capabilities [10].

H5: Consumption of content‑creator or entertainment channels is negatively associated with work competencies among youth.

Participation in online discussions, where the environment is often unmoderated, frequently lacks structured modeling and constructive feedback—both fundamental to self‑efficacy development [33]. Without the mastery validation essential to efficacy strengthening [19], chaotic or argumentative environments may disrupt confidence in one’s communicative or reflective abilities. Irregular reinforcement reduces retention, weakens self‑regulation, and impedes the conversion of observation into competence [10]. Over time, reduced confidence and inconsistent peer support hinder the consolidation of transferable professional skills [12]. This aligns with SCCT’s logic that environments low in reinforcement and constructive social modeling fail to cultivate sustained efficacy or skill performance [21], explaining the negative association between unstructured online‑forum participation and work‑readiness competencies among youth.

H6: Online‑forum participation is negatively associated with work competencies among youth.

**Fig 1:**
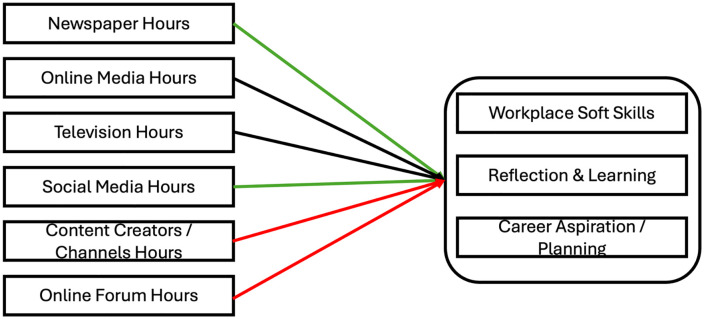
Illustration of the proposed Hypothesis.

The conceptual model in Fig 1 visualizes the proposed relationships between various media types and youth work competencies. It illustrates how different modes of media use may influence the development of self‑efficacy and professional skills, consistent with the learning and environmental reinforcement principles of SCCT. Structured, feedback‑rich, and cognitively engaging media—such as newspapers, online informational sources, and educational television—are expected to foster stronger competencies by providing mastery experiences, meaningful feedback, and reinforcement. In contrast, less structured or passive forms of media interaction—such as unmoderated social media use, entertainment‑driven content, or unregulated online discussions—may hinder the acquisition and expression of work‑related capabilities by offering inconsistent feedback and limited opportunities for skill validation. Collectively, the model presents a comprehensive framework for understanding how diverse media environments contribute to youth employability, professional development, and readiness in a rapidly evolving digital society.

## 3. Methodology

### 3.1. Participants recruitment

This study employed a cross-sectional survey design using a self-administered online questionnaire hosted on Qualtrics, conducted between 3 October and 10 December 2024. The target population comprised tertiary students in Hong Kong who were expected to enter the workforce within one to three years. Focusing on current university students—rather than graduates—allowed the research to capture their work-readiness during their transition into employment, providing a real-time understanding of their aspirations and preparedness before full entry into the labour market. To ensure contextual relevance, only Hong Kong permanent residents were included, while international and mainland Chinese students were excluded, thereby controlling for socio-cultural and economic factors specific to the local population. The sampling strategy was designed to reflect the diversity of Hong Kong’s tertiary education sector, encompassing both government-subsidised and self-financed institutions. Six institutions were randomly selected: three UGC‑subsidised universities (The Chinese University of Hong Kong, The Education University of Hong Kong, and The University of Hong Kong) and three self‑financed institutions (the Hong Kong Institute of Vocational Education, The Hang Seng University of Hong Kong, and The University of Hong Kong School of Professional and Continuing Education).

This selection ensured a relatively balanced representation across institutional types, capturing variation in funding models, academic structures, and student demographics. Questionnaires were distributed via institutional mailing lists with administrative assistance from staff members at participating institutions. The final sample comprised 363 valid and complete responses. Including both publicly funded and self‑financed institutions enhanced representativeness, allowing for meaningful analysis of differences in work-readiness across diverse educational and socioeconomic backgrounds. Ethical approval for this study was obtained from the Survey and Behavioral Research Ethics Committee (SBREC) at The Chinese University of Hong Kong (Reference No.: SBRE 24‑0200). All participants were aged 18 years or above and provided informed consent electronically before completing the survey. Participation was voluntary and anonymous and adhered to institutional data-protection guidelines. No identifying information such as names, phone numbers, or identity numbers was collected. The full dataset is available in S1 Data.

### 3.2. Research design and analytical framework

This study employs a cross‑sectional analytical design to examine the relationships between media consumption and work competencies among tertiary students in Hong Kong. Grounded in SCCT, the framework conceptualizes media as learning environments that shape skill acquisition through observation, feedback, and cognitive engagement. Given the nature of the data, the analysis focuses on correlational associations rather than causal relationships, acknowledging potential endogeneity or reverse causality. The study investigates how different types of media—newspapers, online media, television, social media, content‑creator platforms, and online discussion forums—are associated with three dimensions of youth work competencies: workplace soft skills, reflection and learning, and career aspiration and planning. Through this approach, the study aims to provide an integrated understanding of how structured and feedback‑rich media environments support competence development, while unstructured or passive media contexts may limit reinforcement and hinder professional growth, consistent with SCCT’s emphasis on environmental influences in self‑efficacy and competency formation.

#### 3.2.1. Measurement of the variables.

The first part of this study provides an overview of media consumption and the attributes of work competencies. For the media consumption questions, respondents were asked about the number of hours per week they spent on various types of media, including newspapers, online media, television, content creators/channels, and online discussion forums—consistent with existing research that examines media use intensity and exposure patterns among Hong Kong respondents [(e.g., 35)]. This conceptualization aligns with SCCT, which emphasizes that learning environments—such as media contexts—provide observational experiences and reinforcement that shape self‑efficacy and outcome expectations [20,21]. Accordingly, time spent with each media form serves as an environmental exposure indicator, capturing how different types of content and feedback structures may contribute to the development of work‑related competencies. These measures operationalize the environmental learning component of SCCT by approximating the quality and frequency of mediated experiences that support or hinder efficacy‑building and skill acquisition.

Three competency variables were constructed: workplace soft skills, reflection and learning, and career aspiration/planning. The workplace soft skills variable includes three domains—hope (8 items), communication (8 items), and problem‑solving (5 items); reflection and learning comprises empathy (7 items), reflection (7 items), and learning orientation (5 items); and career aspiration/planning consists of career exploration (5 items) and life planning (3 items). Each variable functions as a composite construct, created by averaging responses across its items to capture interrelated but conceptually distinct dimensions of work readiness. Cronbach’s alpha tests for each competency variable and their domains (Tables A1–A3; S1 Appendix) all exceeded 0.6, confirming internal consistency. These combined measures represent coherent dimensions of youth work readiness, including interpersonal effectiveness, reflective capacity, and future‑oriented planning.

The selection and interpretation of these competency variables are grounded in SCCT, which highlights three interrelated cognitive mechanisms—self‑efficacy, outcome expectations, and goal setting—as the foundation for how individuals acquire skills and form beliefs about career progression [20,21]. In this framework, self‑efficacy refers to one’s confidence in performing specific tasks, and outcome expectations denote beliefs about the likely consequences of those tasks; both are internal cognitive appraisals rather than observable behaviors. The present study does not directly measure these cognitive mechanisms but instead captures their behavioral correlates—competencies that reflect applied ability, adaptive learning, and goal‑directed practice developed through experience and feedback. Each competency variable corresponds to a particular aspect of SCCT’s learning process: workplace soft skills mirror domain‑specific self‑efficacy through demonstrated mastery in communication, teamwork, and problem‑solving; reflection and learning represent cognitive processing and feedback integration consistent with the development of adaptive outcome expectations; and career aspiration and planning align with the goal‑setting mechanism by translating perceived efficacy into deliberate action and self‑regulation. Although the full theoretical sequence of SCCT is not empirically measured, the competency variables are conceptually treated as environmentally reinforced outcomes that connect learning experiences to professional development, capturing the applied dimension of SCCT’s efficacy‑building logic.

#### 3.2.2. Associations Between Media Consumption and Attributes of Work Competencies.

To examine the associations between media consumption patterns and work competencies, this study conducts Ordinary Least Squares (OLS) regression analyses, estimating separate models for each of the three competency dimensions: workplace soft skills, reflection and learning, and career aspiration/planning. The objective is to assess how different types of media consumption relate to distinct aspects of work competencies, providing an empirical basis for interpreting media as environmental learning contexts within the framework of SCCT.

Each regression model takes the form of a linear equation, where the competency variable serves as the dependent variable and the independent variables capture weekly hours spent on various types of media—newspapers, online media, television, social media, content‑creator channels, and online discussion forums. Demographic and contextual variables are included as controls to account for potential confounding influences on competency development. These include age, gender, education level, being born in Hong Kong, income level, and participation in employment‑ or education‑related support programmes.

Age is controlled because older respondents may possess greater maturity, experience, and exposure to professional or academic environments that influence skill formation [36]. Gender is included to account for documented differences in media use, self‑efficacy, and vocational preferences [37,38]. Education level captures variation in analytical and reflective capacity associated with academic attainment [36]. Being born in Hong Kong represents cultural familiarity and integration that may shape confidence and professional adaptability [34]. Income level serves as a proxy for socioeconomic status, which influences access to digital tools and skill‑building resources [35,26]. Participation in employment service or career‑education activities controls for structured developmental experiences that could foster skill acquisition independently of media exposure [14]. Although these control variables address observable sources of bias, unmeasured influences—such as personality traits, intrinsic motivation, or family background—may still affect both media engagement and competency outcomes. Hence, the results are interpreted as associational rather than causal. The regression equation for each dependent variable can be expressed as follows:


$$\begin{array}{c} WSS_{\iota }=\alpha_{\text{1}}+\alpha_{\text{2}}Newspaper_{\iota }+\alpha_{\text{3}}OnlineMedia_{\iota }+\alpha_{\text{4}}Television_{\iota }+\alpha_{\text{5}}SocialMedia_{\iota }+\alpha_{\text{6}}OnlineForum_{\iota }+\alpha_{\text{7}}X_{\iota } \end{array}$$ (1)

$$\begin{array}{c} R\&L_{\iota }=\alpha_{\text{1}}+\alpha_{\text{2}}Newspaper_{\iota }+\alpha_{\text{3}}OnlineMedia_{\iota }+\alpha_{\text{4}}Television_{\iota }+\alpha_{\text{5}}SocialMedia_{\iota }+\alpha_{\text{6}}OnlineForum_{\iota }+\alpha_{\text{7}}X_{\iota } \end{array}$$ (2)

$$\begin{array}{c} CAP_{\iota }=\alpha_{\text{1}}+\alpha_{\text{2}}Newspaper_{\iota }+\alpha_{\text{3}}OnlineMedia_{\iota }+\alpha_{\text{4}}Television_{\iota }+\alpha_{\text{5}}SocialMedia_{\iota }+\alpha_{\text{6}}OnlineForum_{\iota }+\alpha_{\text{7}}X_{\iota } \end{array}$$ (3)

where $WSS_{\iota }$, $R\&L_{\iota }$, and $CAP_{\iota }$ refers to the workplace soft skills, reflection and learning, and career aspiration/planning variables, respectively. Meanwhile, $Newspaper_{\iota }$, $OnlineMedia_{\iota }$, $Television_{\iota }$, $SocialMedia_{\iota }$, and $OnlineForum_{\iota }$ refers to the number of hours per week on newspaper, online media, television, social media, and online forum, respectively. Finally, $X_{\iota }$ refers to a set of control variables employed in this regression model. As such, this analytical design enables a comprehensive examination of how structured and feedback‑rich media environments correspond to higher competency levels, whereas unstructured or passive media contexts may constrain professional development. In line with the assumptions of SCCT, the model treats variations in media exposure as reflections of environmental learning opportunities that influence self‑efficacy and, consequently, the acquisition of applied workplace skills.

## 4. Results

### 4.1. Descriptive statistics

The study collected data from 363 valid respondents, all of whom were Hong Kong permanent residents currently enrolled in one of six tertiary education institutions. All participants were tertiary students expected to enter the workforce within one to three years. As shown in Table 1, demographic profile of the respondents provides insights into their socio‑economic and educational backgrounds. The average age of the respondents was 22.7 years (SD = 1.79), reflecting a young population transitioning from education to employment. Approximately 39.7% of respondents were male. In terms of education, 84.8% were enrolled in degree programs, while the remainder were pursuing sub‑degree qualifications such as associate degrees or higher diplomas. A large majority (84.3%) were born in Hong Kong. The average household income level of respondents was 5.93 (SD = 2.28), corresponding to an income range of approximately HKD $25,000–$30,000 per month, indicating a predominantly middle‑class demographic.

**Table 1:** Descriptive Statistics.

Variable	Description:	Mean	SD
Newspapers Hours	The average number of hours per week that the respondents spent reading newspapers.	.3746	1.8226
Online Media Hours	The average number of hours per week that the respondents spent watching online media (e.g., visiting websites).	4.2331	6.8995
Television Hours	The average number of hours per week that the respondents spent watching television.	1.7644	4.1583
Social Media Hours	The average number of hours per week that the respondents spent on using social medias.	9.9118	10.2928
Content Creators/Channels Hours:	The average number of hours per week that the respondents spent watching content creators/ channels.	2.7163	5.8391
Online Forum Hours	The average number of hours per week that the respondents spent watching or participating in online forums.	2.2141	4.2543
Workplace Soft Skills	A composite index measures the reported level of workplace skills, where 1 indicates possessing weak workplace soft skills and 5 indicates possessing strong workplace soft skills. For more information, refer to Table A1.	3.3050	.5611
Reflection and Learning	A composite index measures the reported level of reflection and learning, where 1 indicates possessing weak reflection and learning abilities and 5 indicates possessing strong reflection and learning abilities. For more information, refer to Table A2.	3.3610	.5479
Career Aspiration/ Planning	composite index measures the reported level of career aspiration and planning, where 1 indicates possessing low career aspiration and planning and 5 indicates possessing high career aspiration and planning. For more information, refer to Table A3.	3.0195	.7538
Age	The respondents’ age.	22.6997	1.7888
Male	Whether the respondent is a male (1 = male, 0 otherwise).	.3966	.4898
Education	The respondents’ current education program enrollment (1 = Degree program, 2 = sub-degree program (e.g., associate degree, higher diploma).	.8484	.3590
Born in HK	Whether the respondent is a born in Hong Kong (1 = born in Hong Kong, 0 otherwise).	.8430	.3643
Income Level	The respondents’ monthly household income level in HKD. (1 = $9999 or below, 2 = $10000–14999, 3 = $15,000-$19,999, 4 = $20,000-$24,999, 5 = $25,000-$29,999, 6 = $30,000 = $39,999, $40,000-$49,999, 7 = $40,000-$49,999, 8 = $50,000-$59,999, 9 = $60,000-$79,999, 10 = $80,000 or above).	5.9256	2.2759
Employment Service Groups	A single-item binary question asks whether the respondent had sought help from local groups or volunteering communities that provide employment services for youth (1 = Yes, 0 = No).	.7879	.4094
Work-Education Related	A single-item question asks respondents whether their educational background is related to their ideal industry (1 = very unrelated, 2 = unrelated, 3 = neutral, 4 = related, 5 = very related)	3.4435	1.2740

Respondents’ media consumption patterns were diverse, reflecting the widespread digital engagement typical of Hong Kong youth. On average, participants spent 0.37 hours per week (SD = 1.82) reading newspapers, indicating minimal engagement with traditional print media. By contrast, online media consumption was significantly higher, averaging 4.23 hours per week (SD = 6.90). Television viewing was moderate at 1.76 hours per week (SD = 4.16), while social media use dominated overall media behavior, averaging 9.91 hours per week (SD = 10.29) on platforms such as Instagram, Facebook, and others. Time spent viewing content‑creator or influencer channels (e.g., YouTube) averaged 2.71 hours per week (SD = 5.83), and respondents spent 2.21 hours per week (SD = 4.25) participating in online discussion forums, showing some engagement with interactive, discussion‑based platforms.

The study also examined three key variables reflecting respondents’ work‑readiness competencies: workplace soft skills, reflection and learning, and career aspiration/planning. Respondents reported an average score of 3.31 (SD = 0.56) for workplace soft skills, suggesting moderate proficiency in communication, teamwork, and problem‑solving. The average score for reflection and learning was 3.36 (SD = 0.55), indicating a fair capacity for self‑reflection, empathy, and continuous learning. The career aspiration and planning variable had an average score of 3.02 (SD = 0.75), reflecting moderate clarity regarding career goals and future planning within the sample. Overall, these descriptive statistics provide a comprehensive overview of the respondents’ demographic characteristics, media consumption behaviors, and levels of work‑related competency. This contextualizes the subsequent analysis exploring how different forms of media engagement correspond to variations in youth work‑readiness competencies.

### 4.2. Regression of media consumption on attributes of work competencies

Table 2 presents the results of the regression analysis examining the associations between various forms of media consumption and three dimensions of work competencies: workplace soft skills, reflection and learning, and career aspiration/planning. The findings show distinct but generally modest association patterns across media types and competency domains. For workplace soft skills, newspaper consumption shows a relatively marginal positive association (β = 0.0273, p < 0.1). Although small in magnitude, each additional hour of newspaper reading per week corresponds to an estimated 0.03‑point increase on the five‑point competency scale. Social media usage displays a positive and statistically significant association (β = 0.0097, p < 0.01), indicating that greater engagement on these platforms corresponds with slightly higher reported workplace competencies, particularly in communication and collaboration. In contrast, content‑creator or entertainment channels exhibit a relatively marginal negative association (β = –0.0079, p < 0.1), suggesting that heavier use of entertainment‑oriented content is weakly related to lower skill levels. Online forum participation shows a clear negative association (β = –0.0164, p < 0.01), indicating that additional hours of exposure correspond with small but consistent decreases in reported competencies. Other media types, such as online media and television, show no statistically significant associations with workplace soft skills.

**Table 2:** Determinants of psychological work readiness among the respondents.

	Workplace Soft Skills	Reflection and Learning	Career Aspiration/ Planning
Newspapers Hours	.0273* (.0140)	.0385*** (.0145)	.0342* (.0212)
Online Media Hours	.0019 (.0042)	.0019 (.0043)	−.0061 (.0063)
Television Hours	.0037 (.0068)	.0029 (.0070)	.0064 (.0102)
Social Media Hours	.0097*** (.0028)	.0085*** (.0028)	.0064 (.0042)
Content Creators/Channels Hours:	−.0079* (.0044)	−.0058 (.0046)	−.0066 (.0068)
Online Forum Hours	−.0164*** (.0063)	−.0131** (.0065)	−.0169* (.0095)
Age	−.0093 (.0153)	−.0075 (.0158)	.0219 (.0230)
Male	.0827 (.0529)	.0332 (.0547)	.1387* (.0798)
Education	.3128 (.0816)	.2695*** (.0844)	.1969 (.1232)
Born in HK	−.0714 (.0708)	−.0508 (.0732)	−.0423 (.1068)
Income Level	.0262** (.0114)	−.0032 (.0118)	.0307* (.0172)
Employment Service Groups	.0710*** (.0205)	.0410* (.0212)	.1241*** (.0309)
Work-Education Related	.3152*** (.0747)	.2624*** (.0773)	.2315** (.1127)
Constant	2.5667*** (.3859)	2.9361*** (.3990)	1.5392*** (.5823)
N	363	363	363
R^2^	.2879	.2012	.1015

*p < .1, **p < .05, ***p < .01. Standard errors are in the parathesis.

For reflection and learning, newspaper consumption is positively associated (β = 0.0385, p < 0.01), representing the strongest effect observed among the media variables. This corresponds to an approximate 0.04‑point increase per additional weekly hour of reading. Social media use is also positively associated (β = 0.0085, p < 0.01), indicating small but steady improvements in reflective and learning competencies as engagement increases. Conversely, online forum use presents a negative association (β = –0.0131, p < 0.05), showing that frequent participation in loosely structured or argumentative online spaces is associated with modest reductions in reflective learning capacity. Other media categories—including television, online media, and content‑creator channels—do not display statistically significant relationships with reflection and learning. Likewise, for career aspiration and planning, newspaper consumption again shows a relatively marginal positive association (β = 0.0342, p < 0.1). The coefficient suggests an incremental increase of just over 0.03 points for each additional hour of newspaper reading, reflecting a small but consistent directional link with goal setting and life planning. In contrast, online forum usage exhibits a relatively marginal negative relationship (β = –0.0169, p < 0.1), implying slightly lower career planning scores among individuals more engaged in online discussions. Other media forms—including online media, television, social media, and content‑creator channels—show no significant associations with career aspiration and planning.

Among the control variables, education level reveals a robust positive association with workplace soft skills (β = 0.3128, p < 0.01) and reflection and learning (β = 0.2695, p < 0.01), representing differences of roughly 0.27–0.31 points between degree‑ and sub‑degree‑level respondents. Participation in employment service or work‑related programmes is positively related to all three competency dimensions, including workplace soft skills (β = 0.0710, p < 0.01) and career aspiration/planning (β = 0.1241, p < 0.01). Household income is positively related to workplace soft skills (β = 0.0262, p < 0.05) and career aspiration/planning (β = 0.0307, p < 0.1), although the latter effect is also relatively marginal. Finally, male respondents report slightly higher career aspiration and planning scores (β = 0.1387, p < 0.1), again a relatively weak association, while gender has no significant effects on workplace soft skills or reflection and learning.

Overall, the results suggest that structured and feedback‑rich media—notably newspapers and purposeful social media engagement—are positively associated with youth work competencies, though the magnitude of these relationships remains modest. Several associations, particularly those significant at the p < 0.1 level, should be viewed as relatively marginal and interpreted with caution. In comparison, unstructured or entertainment‑driven media, such as unmoderated online forums or influencer channels, show weak negative or null associations with competency development. Taken together, these patterns indicate that both media structure and demographic factors—including education, income, and participation in employment support programmes—correspond with variations in work‑readiness among Hong Kong tertiary students, albeit with small overall effect sizes.

## 5. Discussion and conclusion

### 5.1. Discussion of media consumption and work competency development

The results reveal consistent associations between media consumption and youth work competencies. In line with SCCT, the findings highlight that structured and feedback‑rich environments facilitate skill acquisition through observation, reinforcement, and purposeful engagement. Media that provide such characteristics—like newspapers and professionally oriented social media—are positively related to skill formation because they promote attention, critical thinking, and self‑efficacy development [19,11]. In contrast, unstructured or passive media—including television, general online browsing, and entertainment‑driven platforms—show weak or negative associations with work competencies, reflecting fewer opportunities for practice and meaningful feedback [10,12]. Overall, the results confirm that the quality of engagement—rather than time spent—determines how effectively media environments reinforce competency development, consistent with SCCT’s emphasis on feedback‑driven mastery experiences.

Among the media examined, traditional print media remain the most structured and cognitively reinforcing. Newspaper reading demands sustained attention, analytical reasoning, and reflection [19]; through such discipline, readers engage deliberately with complex information and internalize professional discourse patterns [11]. These experiences strengthen communication confidence and self‑regulatory learning, aligning with SCCT’s view that recurring mastery opportunities build efficacy. Accordingly, H1 is supported, showing that sustained print engagement contributes to work readiness among Hong Kong youth. By contrast, general online browsing shows no significant relationship with work competencies. Although information access is high, it often lacks the structure or reinforcement required for efficacy building [10,12]. Online environments characterized by fragmented, algorithmically generated content tend to encourage surface‑level engagement rather than feedback‑driven learning, constraining opportunities for adaptive competence [27]. Consequently, H2 is supported, indicating that uncurated digital consumption provides a weak basis for applied learning and skill growth.

A similar pattern appears for television, which also shows no significant link to competencies. This medium’s one‑way mode of communication offers limited potential for the reinforcement and validation processes through which efficacy typically develops [33]. While television may provide occupational imagery, it rarely enables direct interaction or feedback. Hence, H3 is supported, confirming that passive and unidirectional media contribute little to work‑related competency development. As such, social media platforms present a more dynamic setting. When used constructively, they offer interactive spaces where users can observe, model, and exchange performance feedback—conditions favorable to SCCT’s principles of vicarious learning and social persuasion [19,34]. Purposeful engagement, such as participating in professional networks and collaborative learning communities, enhances workplace soft skills and reflection through dialogue and peer reinforcement. Yet, when use is unstructured or primarily recreational, these benefits diminish because reinforcement and goal orientation decrease [10]. Thus, H4 is partially supported: social media use may enhance competencies when engaged for learning and collaboration, but not when centered on entertainment.

Entertainment‑oriented or creator‑based platforms show weaker or negative relationships with competencies. While H5 anticipated broader negative effects, the evidence indicates significance only for workplace soft skills. Such spaces portray models of success but rarely provide mastery verification or credible feedback. In SCCT terms, popularity metrics substitute authentic reinforcement, providing limited efficacy support [10,34]. The absence of significant effects on reflective or planning competencies suggests that higher‑order cognitive skills rely more on structured settings like education or mentorship, which offer clearer mastery opportunities. Any negative impacts from entertainment media likely stem from time displacement rather than direct erosion of cognitive ability [39]. Therefore, H5 is partially supported: greater exposure to entertainment content correlates with lower soft‑skill strength. Similarly, unmoderated online forums show negative associations with workplace soft skills (H6 partially supported). These environments often lack structure, civility, and constructive feedback, limiting the reinforcement processes that promote communicative efficacy [10,12]. Without consistent peer support or positive modeling, interaction quality deteriorates, reducing users’ confidence in effective participation. Reflection and planning competencies are less affected, as these depend more on individual cognitive processing than on social validation.

Overall, the findings demonstrate that not all media foster learning in equal measure. Structured, cognitively demanding, and feedback‑oriented environments help youth apply observation, reinforcement, and self‑reflection to their skill development—mechanisms that SCCT identifies as essential to efficacy. Conversely, unstructured or entertainment‑driven contexts restrict these processes by offering little reinforcement or structured modeling. Although effect sizes are modest, the consistency across competencies underscores that environmental structure and engagement quality shape how youth acquire communication, reflection, and planning skills. Thus, everyday media serve as informal yet meaningful learning arenas that influence how young people gain self‑efficacy, adaptive capacity, and work‑readiness in an increasingly digital society [11,12].

### 5.2. Implications of the finding

The findings of this study carry significant implications for various groups, including the government, educational institutions, employers, and media platforms. Each has a key role to play in addressing the challenges identified in this research and in building a more coordinated ecosystem that supports the development of core work competencies among young people in Hong Kong. Strengthening these collaborations is essential to ensure that skill development keeps pace with the rapidly evolving media landscape and the demands of a knowledge‑based economy. For the government, promoting digital literacy and raising awareness about productive media consumption should be a policy priority. The study highlights how structured media use—such as reading newspapers and engaging with professional social media platforms—can positively influence key work‑readiness skills, including communication, reflection, and problem‑solving. Public campaigns could therefore encourage young people to engage with media purposefully for professional growth while discouraging overreliance on unstructured or entertainment-driven platforms that offer limited reinforcement for learning. In tandem, policymakers should strengthen programs that help youth cultivate practical employability skills—such as communication, teamwork, and planning—through career‑education initiatives, mentorship schemes, and experiential learning opportunities. Expanding training and development programmes, especially for underprivileged or at‑risk youth, would further help narrow skill disparities and improve overall workforce preparedness.

These governmental efforts would be most effective when integrated with educational strategies. Educational institutions hold a central role in helping students transform media engagement into practical competencies for employment. Universities and colleges could incorporate media literacy and digital professionalism into their curricula, equipping students with the critical capacity to assess, interpret, and apply media content for career development. Strengthening career counseling services and organizing structured training workshops would assist students in clarifying professional goals and developing soft skills crucial for workplace success. Furthermore, closer collaboration between academia and industry could provide mentorship and networking opportunities that expose students to real‑world professional standards and practices. Complementing these measures, reflective learning activities—such as self‑assessment, empathy development, and journaling—would help students cultivate adaptive thinking and interpersonal awareness, both of which are indispensable in dynamic work environments. Employers and industry leaders also play an integral role in bridging the transition from education to employment, reinforcing and extending the skills acquired through formal learning. By investing in youth development initiatives and training programmes designed in partnership with educational institutions, employers can help align academic preparation with industry expectations. Providing internships, project‑based placements, apprenticeships, and mentorship opportunities enables young people to gain hands‑on experience and refine essential competencies such as teamwork, communication, and problem‑solving. Employers can further support productive media engagement by encouraging employees and prospective hires to use professional networking platforms—such as LinkedIn—to exchange insights, participate in professional learning communities, and expand their knowledge networks, thus transforming media interaction into a tool for continuous professional growth.

At the same time, media and technology platforms share responsibility for cultivating environments that promote constructive, educational, and reflective engagement. Social media companies and content creators could be incentivized to produce or highlight content that supports employability, professional communication, and lifelong learning. Collaboration with educational institutions could help design digital initiatives that blend active engagement with structured feedback and reflection. Platform moderators should likewise foster spaces that encourage purposeful interaction and constructive dialogue, ensuring that youth media use contributes positively to skill building rather than distraction. Taken together, these efforts underscore that enhancing youth work readiness requires coordinated action across sectors. By aligning educational reform, policy initiatives, workplace training, and digital governance, Hong Kong can create an integrated ecosystem that nurtures young people’s capacity for communication, reflection, problem-solving, and planning. In doing so, the city can strengthen the foundations of a resilient, future‑ready workforce prepared to thrive in an increasingly media‑saturated and knowledge‑driven economy.

### 5.3. Limitations and future research work

While this study provides valuable insights into the relationships between media consumption and work competencies among Hong Kong youth, several limitations should be acknowledged. Addressing these limitations in future research could provide a more comprehensive understanding of how media environments contribute to youth skill development and work readiness. First, the study employs a cross‑sectional design, which limits the ability to establish causal relationships between media consumption and competency development. Although the results highlight significant associations, the cross‑sectional and observational nature of the data raises concerns about potential selection bias and endogeneity. Selection bias may occur if youth who choose to participate in the survey differ systematically from those who do not, potentially limiting the representativeness of the sample. More importantly, endogeneity concerns arise because the observed relationships may be subject to reverse causality or omitted variable bias. For instance, individuals with pre‑existing levels of confidence or motivation to improve their work skills may selectively consume more structured media or invest more time in professional learning activities, making it difficult to determine whether media consumption influences skill formation or vice versa. Similarly, unobserved factors such as family socioeconomic background, personality traits, or prior success experiences may simultaneously affect both media use and competency development, creating spurious associations. Although this study controls for observable demographic and contextual characteristics, these controls cannot fully eliminate endogeneity. Future research could address these limitations through longitudinal designs that track changes in media consumption and skill development over time, helping to establish temporal ordering and reduce concerns regarding reverse causality.

Second, the institutional composition of the sample presents an important methodological consideration. The inclusion of students from both elite universities (e.g., CUHK, HKU) and lower‑ranked or self‑financed institutions (e.g., Hang Seng University of Hong) introduces potential omitted‑variable bias. Academic selectivity, prior achievement, and socioeconomic background likely shape both media consumption patterns and the development of work competencies. Institutional tier may therefore function as an upstream determinant influencing both the independent and dependent variables [40]. Without adequately controlling for university affiliation or proxies for academic performance, the observed relationships may partially reflect compositional differences rather than substantive effects of media use. The survey also did not collect data on university affiliation, grade point average, or academic selectivity, preventing direct statistical control for institutional tier or student performance. In this context, institutional divergence should be treated as theoretically central, and future research should incorporate these indicators or apply within‑strata analyses to disentangle structural and individual effects on the observed media–competency relationships.

Beyond institutional factors, a broader issue concerns the role of social origins and cultural capital in shaping both media practices and competency development. Scholarship on cultural consumption and inequality suggests that social status strongly predicts patterns of media use, cognitive style, and communicative competence [41,42]. If this proposition holds true in Hong Kong, social background may operate not merely as a control variable but as a mechanism linking media engagement and skill acquisition. However, this study did not measure participants’ parental education, occupational status, or other indicators of social class, limiting analysis of how structural origins might condition media use or skill formation. Future studies could extend the model by testing whether cultural consumption mediates the relationship between socioeconomic status and competency development—offering a theoretical bridge between media engagement, social reproduction, and inequality. Although the current dataset precludes such an analysis, this remains a promising direction for understanding how media can reproduce or mitigate social advantage among youth.

Third, the study focuses exclusively on tertiary students in Hong Kong. While this provides valuable insights into a critical transitional group poised to enter the workforce, it limits the generalizability of the findings to other youth populations—such as those not pursuing higher education, vocational trainees, or young adults already in employment. Future research could broaden its scope to include youth from diverse educational and occupational backgrounds to develop a more holistic understanding of how media consumption relates to employability and career preparedness across different segments of the Hong Kong youth population. Fourth, the measurements of media consumption used in this study rely on self‑reported data, which may be subject to recall and social‑desirability biases, leading to possible under‑ or over‑estimation of media exposure. Future research could employ objective measures, such as digital usage tracking tools or data directly obtained from media platforms, to generate more accurate and reliable estimates of media behaviors. Additionally, the integration of qualitative approaches—such as interviews, diaries, or focus groups—could complement quantitative analyses by yielding deeper insights into how young people perceive and apply learning from different media environments in their skill‑building processes.

Fifth, the study does not examine the specific nature or quality of content consumed within each media category, even though different kinds of content may have distinct effects on learning outcomes. For example, engagement with educational, civic, or professional content on digital platforms may enhance reflection and problem‑solving, while purely entertainment‑oriented content may not. Future research should therefore examine content typologies and their relationship to competency development, identifying the types of media experiences that most effectively support employability and professional growth. This approach could inform the development of targeted media literacy initiatives and educational interventions that maximize positive learning outcomes. Sixth, while the study controls for several demographic and contextual variables, additional unmeasured factors may influence work‑related skill development. Personality traits, family background, peer dynamics, and access to technological or social resources may all interact with media use to shape learning and efficacy outcomes. Due to practical constraints on survey length, not all of these variables could be included. Future studies should consider incorporating such factors, either through expanded quantitative models or complementary qualitative analyses, to capture the complex interplay between individual, social, and structural influences on youth competency development.

Seventh, although this study examined associations between different forms of media consumption and work competencies, it did not formally test whether these competencies serve as mediators linking media engagement to real‑world employment outcomes. Future research could extend this framework by employing longitudinal, multi‑wave, and mediation models—such as structural equation modeling (SEM), cross‑lagged panel models, or hierarchical path analysis—to evaluate how competencies evolve over time and how they function as mediating variables that connect media usage patterns with eventual employment outcomes, such as job attainment, workplace performance, career adaptability, or job satisfaction. Such an approach would allow researchers to assess both direct and indirect pathways of influence, testing whether structured and feedback‑rich media environments contribute to professional success primarily through their effect on skill formation and self‑efficacy. Longitudinal evidence would also make it possible to determine whether certain media consumption habits serve as sustained learning mechanisms that reinforce professional growth, while others may hinder progression through distraction or time displacement. By modeling competencies as mediating mechanisms within long‑term employability outcomes, future studies could provide stronger empirical support for the theoretical proposition that purposeful media engagement fosters lasting work readiness and career success.

Lastly, while this study draws on Social Cognitive Career Theory (SCCT) as its theoretical foundation, it does not directly measure certain core cognitive constructs—such as self‑efficacy beliefs (confidence in one’s ability to perform tasks) and outcome expectations (beliefs about the consequences of performing those tasks). Instead, the study operationalizes work readiness through behavioral indicators, including communication, problem‑solving, and career planning skills—observable capacities that reflect applied competence rather than internal cognitive beliefs. Although SCCT posits that behavioral competencies develop in conjunction with self‑efficacy and outcome expectations, these underlying cognitive elements were not directly measured. Future research could incorporate validated instruments—such as scales assessing career decision‑making self‑efficacy and outcome expectations—alongside behavioral measures. Doing so would allow researchers to distinguish between belief‑based and performance‑based dimensions of competence and to more comprehensively evaluate the theoretical pathways proposed by SCCT.

In summary, future research should build upon the current findings by adopting longitudinal, mixed‑method, and theoretically integrated approaches that explore how media engagement contributes to skill acquisition, professional readiness, and real employment outcomes. Such work would not only refine understanding of media’s role in learning and development but also strengthen the empirical foundation for designing interventions that enhance youth employability in a media‑saturated world.

## Supporting information

S1 Appendix Detailed measurement constructs and reliability statistics for all survey instruments, including workplace soft skills (Cronbach’s α = 0.8006), reflection and learning (Cronbach’s α = 0.6975), and career aspiration/planning (Cronbach’s α = 0.7162). (DOCX)

S1 Data Underlying cleaned dataset used for the analyses presented in this study. (XLSX)
