# Supplementary material for: Media consumption and youth work competencies: Evidence from the Hong Kong youth survey
Source: PLoS One. 2026 May 14;21(5):e0349087. doi: 10.1371/journal.pone.0349087 (PMC13175486; doi:10.1371/journal.pone.0349087)
Supplement: S1 Appendix — (DOCX) [file pone.0349087.s001.docx]

Table A1: Workplace Soft Skills (Cronbach Alpha: 0.8006)

| Domain: | Question | Cronbach Alpha (Workplace Soft Skill) | Cronbach Alpha  (Items) |
| --- | --- | --- | --- |
| Hope |  | 0.6710 |  |
|  | I can think of many ways to get out of a jam. |  | 0.8735 |
|  | I energetically pursue my goals. |  | 0.8762 |
|  | There are lots of ways around any problem. |  | 0.8727 |
|  | I can think of many ways to get the things in life that are important to me. |  | 0.8744 |
|  | Even when others get discouraged, I know I can find a way to solve the problem. |  | 0.8690 |
|  | My past experiences have prepared me well for my future. |  | 0.8781 |
|  | I’ve been pretty successful in life. |  | 0.8852 |
|  | I meet the goals that I set for myself. |  | 0.8815 |
| Communication: |  | 0.7116 |  |
|  | I prepare for all communications and think things through before I speak. |  | 0.7832 |
|  | I find it easy to listen to what other people have to say without interrupting. |  | 0.7878 |
|  | I am confident when I talk to people and speak clearly without mumbling. |  | 0.7720 |
|  | I am good at getting my point across in a clear, concise manner without waffling. |  | 0.7808 |
|  | I find it easy to concentrate on what others are saying and don’t lose my focus. |  | 0.7707 |
|  | I can keep my cool when talking to other people even if I feel angry about what they say. |  | 0.7836 |
|  | I don’t shout and point at people when we have a heated conversation. |  | 0.7792 |
|  | I write down what the other person says in order to understand them accurately. |  | 0.8117 |
| Problem Solving |  | 0.6721 |  |
|  | Given enough time and effort, I believe I can solve most problems that confront me. |  | 0.7246 |
|  | When faced with a novel situation I have confidence that I can handle problems that may arise. |  | 0.7144 |
|  | I trust my ability to solve new and difficult problems. |  | 0.6968 |
|  | I have the ability to solve most problems even though initially no solution is immediately apparent. |  | 0.6980 |
|  | When confronted with a problem, I am unsure of whether I can handle the situation. (R) |  | 0.8936 |
| Teamwork |  | 0.8826 |  |
|  | I think that teamwork is important. |  | 0.7620 |
|  | People who work in teams can learn more than if they work by themselves. |  | 0.7786 |
|  | I feel confident in my ability to work in a team. |  | 0.7350 |
|  | I know how to give my team members feedback that will not hurt their feelings. |  | 0.7522 |
|  | I am good at communicating with my team members. |  | 0.7555 |
|  | I ask others for feedback. |  | 0.7789 |

All questions are measured on an ordinal scale ranging from 1 (Strongly Disagree) to 5 (Strongly Agree). Items marked with (R) are reverse-coded.

Table A2: Reflection and Learning (Cronbach Alpha: 0.6975)

| Domain: | Question | Cronbach Alpha (Reflection and Learning) | Cronbach Alpha  (Items) |
| --- | --- | --- | --- |
| Empathy |  | 0.7784 |  |
|  | Before criticizing somebody, I try to imagine how I would feel if I were in their place. |  | 0.6997 |
|  | If I'm sure I'm right about something, I don't waste much time listening to other people's arguments. (R) |  | 0.6327 |
|  | I sometimes try to understand my friends better by imagining how things look from their perspective. |  | 0.5572 |
|  | I believe that there are two sides to every question and try to look at them both. |  | 0.6044 |
|  | I sometimes find it difficult to see things from the other person's point of view. (R) |  | 0.6649 |
|  | I try to look at everybody's side of a disagreement before I make a decision. |  | 0.6057 |
|  | When I'm upset at someone, I usually try to "put myself in their shoes" for a while. |  | 0.6132 |
| Reflection: |  | 0.6243 |  |
|  | I love exploring my inner self. |  | 0.8816 |
|  | I often love to look at my life in philosophical ways. |  | 0.8768 |
|  | I love to meditate on the nature and meaning of things. |  | 0.8784 |
|  | My attitudes and feelings about things fascinate me. |  | 0.8837 |
|  | I love analyzing why I do things. |  | 0.8864 |
|  | People often say I’m a deep, introspective type of person. |  | 0.8933 |
|  | I’m very self-inquisitive by nature. |  | 0.8798 |
| Learning Orientation: |  | 0.6467 |  |
|  | I enjoy learning about new topics. |  | 0.8309 |
|  | I like to read about diverse topics. |  | 0.8465 |
|  | I find pleasure in learning. |  | 0.8459 |
|  | I am intrinsically motivated to constantly expand my knowledge. |  | 0.8355 |
|  | I seek deep conceptual knowledge to complete the task assigned to me. |  | 0.8548 |

All questions are measured on an ordinal scale ranging from 1 (Strongly Disagree) to 5 (Strongly Agree). Items marked with (R) are reverse-coded.

Table A3: Career Aspiration/ planning (Cronbach Alpha: 0.7162)

| Domain: | Question | Cronbach Alpha if item deleted for multi-item for variables (Career Aspiration/ planning) | Cronbach Alpha if item deleted for multi-item for variables. (Items) |
| --- | --- | --- | --- |
| Clear Career Exploration: |  | 0.7321 |  |
|  | I am sure that I know the type of job that is best for myself. |  | 0.8631 |
|  | I am sure that I know the type of organization I want to work for. |  | 0.8512 |
|  | I am sure that I know exactly the occupation I want to enter. |  | 0.8524 |
|  | I am sure of my preference for a specific organization. |  | 0.8708 |
|  | I am sure of my preference for a specific position. |  | 0.8538 |
| Life Planning |  | 0.6989 |  |
|  | I fully understand the concept of career and life planning. |  | 0.6557 |
|  | I truly believe in the importance of career and life planning. |  | 0.7518 |
|  | I know how to plan for my future. |  | 0.6217 |

All questions are measured on an ordinal scale ranging from 1 (Strongly Disagree) to 5 (Strongly Agree). Items marked with (R) are reverse-coded.
